# Spectrometric Prediction of Nitrogen Content in Different Tissues of Slash Pine Trees

**DOI:** 10.34133/2022/9892728

**Published:** 2022-01-12

**Authors:** Yanjie Li, Honggang Sun, Federico Tomasetto, Jingmin Jiang, Qifu Luan

**Affiliations:** ^1^Research Institute of Subtropical Forestry, Chinese Academy of Forestry, Hangzhou, Zhejiang 311400, China; ^2^AgResearch Ltd., Christchurch 8140, New Zealand

## Abstract

The internal cycling of nitrogen (N) storage and consumption in trees is an important physiological mechanism associated with tree growth. Here, we examined the capability of near-infrared spectroscopy (NIR) to quantify the *N* concentration across tissue types (needle, trunk, branch, and root) without time and cost-consuming. The NIR spectral data of different tissues from slash pine trees were collected, and the *N* concentration in each tissue was determined using standard analytical method in laboratory. Partial least squares regression (PLSR) models were performed on a set of training data randomly selected. The full-length spectra and the significant multivariate correlation (sMC) variable selected spectra were used for model calibration. Branch, needle, and trunk PLSR models performed well for the *N* concentration using both full length and sMC selected NIR spectra. The generic model preformatted a reliable accuracy with R^2^_C_ and R^2^_CV_ of 0.62 and 0.66 using the full-length spectra, and 0.61 and 0.65 using sMC-selected spectra, respectively. Individual tissue models did not perform well when being used in other tissues. Five significantly important regions, i.e., 1480, 1650, 1744, 2170, and 2390 nm, were found highly related to the *N* content in plant tissues. This study evaluates a rapid and efficient method for the estimation of *N* content in different tissues that can help to serve as a tool for tree *N* storage and recompilation study.

## 1. Introduction

Slash pine, which original from North America, has been widely planted in China for resin and timber production [1, 2]. Research on variation in the amount of *N* allocated to reserves in plant tissues could provide new insights into various aspects of defense and *N* contents with the changing seasons [3].

N, which relates to the protein synthesis, is one the most important macronutrients that support plant growth. Conversely, *N* is also the most common limiting factor for plant and natural ecosystem growth and development. Plants collect and store *N* from soil or atmospheric deposition as a reserve to maintain metabolism for growth [4]. Internal *N* cycling allowed the plant store *N* themselves for tree growth [5, 6]. However, the internal cycling amount of *N* storage and consumption has been less studied, for example, when it starts to reserve [4], how it is related to the environmental factors such as season and soil fertility [7], how *N* affects the ecophysiology process and contributes to different plant tissues for tree growth, [8], and how the variation of *N* storage and consumption amounts in different plant tissues.

Therefore, it is necessary and helpful to understand the internal cycling process of *N* in trees for plant ecology and management. However, the dearth of research on the variation of *N* storage and consumption is mainly ascribed to the cost and time-consuming methods, e.g., a large number of samples need to be considered for multiple plant species and methods such as atomic absorption spectrometry [9, 10] and chromatography [11] need to be deployed.

Near-infrared spectroscopy (NIRS) has been wildly used for plant chemical component analysis [12–16]. The C-H, N–H, and O–H bonds have a strong sensitivity in the range of NIR spectroscopy, which could vibrate, stretch, and bend when interacting with NIR spectra [17]. PLSR [18] has been highly used to predict the chemical content using NIR spectra. However, the application of NIR spectroscopy in analyzing the changes in *N* content in different tree tissues is poorly understood. It is mainly concentrated on leaves under different environmental conditions [19–21].

Here, we hypothesis that (1) the reflectance spectroscopy is feasible to characterize the *N* content in different tree tissues using the PLSR model, (2) the predictability is decreased from one tissue models to another, (3) the most important regions of absorption features associated with *N* content can be found using sMC variable selection, and more importantly, (4) generic (mixed tissue) calibration models can be used for predicting the variation of *N* content across tree tissues.

## 2. Materials and Methods

### 2.1. Sampling

A considerable variation among genotypes of plant and environment is needed to get an accurate model [22]. As such, during summer and winter 2016, we collected samples from the slash pine experimental plantation at Tianmu Mountains region in Hangzhou, China (30°42′N, 120°30′E). This experimental plantation was established in January 1993 at three sites (each site was planted with a distance of 2.0 m between trees). Each site contained 30 rows, in each row, 6 trees were planted. Needle, branch, trunk, and root samples were collected from three or four mature individuals per slash pine. Fresh and new needles and branches were collected from the upper crown using a 20-meter-high pruning shear. From each trunk, a 8 cm × 4 cm stem phloem was extracted at the breast height. Root samples were collected from the coarse and fine roots with equal weight (20 g). All tissue samples were stored at -4°C and shipped to the laboratory to avoid respiration. Subsequently, samples were dried in oven at 60°C and then ground into powder using a Wiley mill fitted with a 2 mm screen, which make the powder have a consistent size smaller than 2 mm. Each sample was then stored into a tube container. A total of 1985 tree samples were collected, and four tissues were collected from each tree, which makes 1985 samples for each tissue. In total, 7940 samples were collected.

### 2.2. Spectral Collection

All of the tissue samples were placed into an air-dried room for a consistence of moisture content (~12%). Then, the powder of each sample from all tissues was used for NIR reflectance spectra collection using a NIR spectrometer (LF-2500, Spectral evolution, USA) with a 10 mm diameter contact fiber-optical probe. The probe was firstly placed on a standard whiteboard for calibration; for stability reasons, the probe was directly placed onto the powder surface using a holder while collecting spectra. The maximum depth of each powder was ~3 cm to make sure the light could not penetrate the sample. The length of spectra was ranged from 1100 to 2500 nm with a 8 nm resolution (totaling 175 wavelengths). The final absorbance spectrum (log 1/reflectance) was the average value of 32 scan times [23, 24].

### 2.3. N Content Analysis

All of the samples were analyzed for *N* content using a total nitrogen analyser (TNM-1, Shimadzu, Columbia, MD) based on the Kjeldahl method [25] and calculated according to Gebauer et al. [26]. The *N* content was reported here as the percentage of dry mass.

### 2.4. Model Calibration and Validation

PLSR models, which is one of the most common methods for model calibration based on spectral data [27, 28], were generated to predict *N* content (% dry mass) of the needle, branch, trunk, root tissues, and a mixture of all tissues together. Spectra data were first processed using standard normal variation (SNV) and first derivative using Savitzky-Golay smoothing (window size:15 data points) [29] which increased result accuracy when comparing with other types of preprocessing methods taken into consideration in our study. For each plant tissue calibration, each model was run 200 times for performance evaluation. The dataset was split into 80% for training and 20% for testing. During the lace of no external validation on an independent test set that was collected in a different year or similar condition. To overcome this limitation, we performed a blockwise cross validation that was named test set validation, and the results were presented as calibration and cross-validation results. The training data were used for model train using leave-one-out cross-validation (LOO). The model performance was tracked by using the coefficient of determination and that root-mean-square error derived from calibration (R^2^_C_ and RMSE_C_) and cross-validation (R^2^_CV_ and RMSE_CV_). We used sMC (*α* = 0.05) [30] as a variable selection method to find out the best PLSR model performance with less spectral variables. This sMC-PLSR method could minimize the effect of irrelevant spectra variables and highlight the most relevant variables that respond to chemical components [31–33]. Optimal latent variables were selected for each PLSR model to obtain the best model. The pls package [34] was used for PLSR and sMC-PLSR model calibration, and the plsVarSel [35] was used for sMC variables selection in R software version 3.1.2; [36].

## 3. Results

### 3.1. The *N* Content Variation

**Figure 1:**
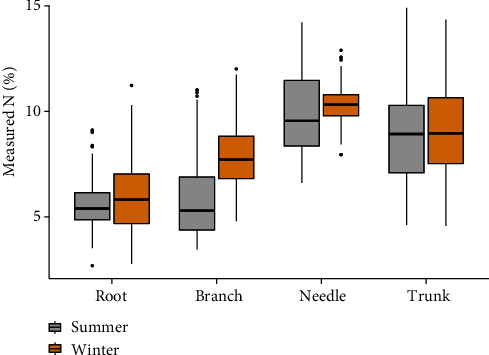
The variation of measured *N* content in different parts of slash pine trees (root, branch, needle, and trunk) in different seasons.

Data of the mixture of all tissues have a reasonable large variation (2.7 to 18.2%). In both summer and winter, root has the lowest *N* content (5.7 and 6.6%, respectively) compared with other tissues. Trunk has the largest *N* content variation, followed by needle and branch tissues. Seasonal variation of *N* content across different tissues is significant especially in branch and needle (Figure 1).

### 3.2. *N* Prediction Using Full Length Spectra

**Figure 2:**
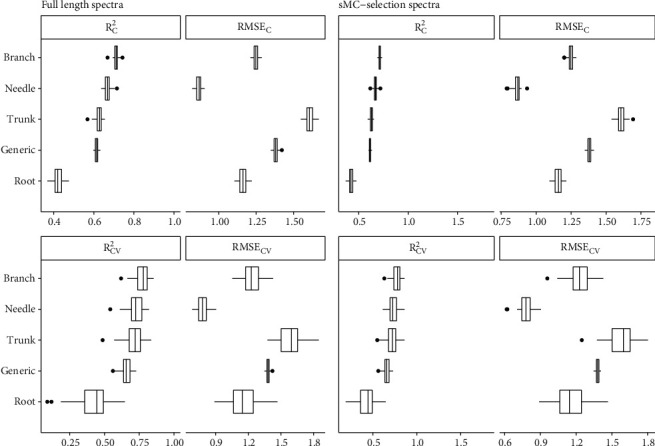
Distribution (95% confidence intervals) of calibration and cross-validation statistics from 100 simulations of models predicting nitrogen content (*N*) in the needle, trunk, branch, and root tissues and the generic of slash pine trees using full-length spectra and sMC-selected spectra. Black vertical line: median value; and the dots represent outliers.

**Figure 3:**
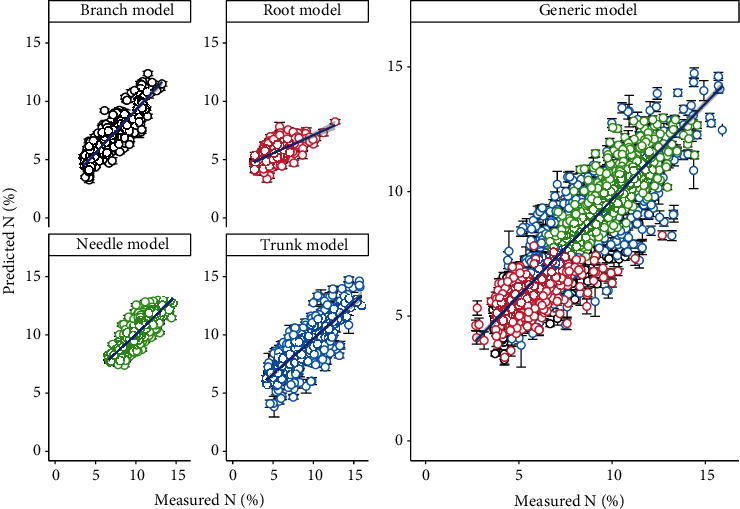
Measured and predicted nitrogen content (*N*) in the needle, trunk, branch, and root tissues and the generic models of slash pines trees using full length of NIR spectra. Error bars: standard deviations of 100 simulated models.

**Figure 4:**
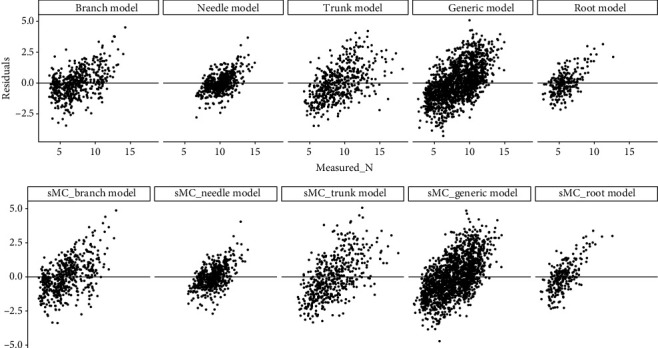
Residuals plotted against measured *N* in needle, trunk, branch, root, and the generic models of slash pine trees using both full-length spectra and sMC-selected spectra.

The *N* content varied over 3-fold in branch and trunk, 2-fold in needle and root, respectively, which could enhanced the prediction accuracy on four types of slash pine tissues using full length NIR spectra, including individual tissues model and generic model (Figures 2(a), 2(c), and 3). Branch model produced the highest mean R^2^_C_ of 0.71 (ranged from 0.67 to 0.74) and R^2^_CV_ of 0.77 (ranged from 0.62 to 0.85), followed by needle model (mean R^2^_C_ and R^2^_CV_ were 0.67 (range: 0.64-0.72) and 0.73 (range: 0.54-0.82), respectively), trunk model (mean R^2^_C_ and R^2^_CV_ were 0.63 (range: 0.57-0.66) and 0.71 (range: 0.49-0.84), respectively), and generic model (mean R^2^_C_ and R^2^_CV_ were 0.62 (range: 0.60-0.64) and 0.65 (range: 0.56-0.73), respectively). The lowest R^2^_C_ and R^2^_CV_ were found in root model, with the mean R^2^_C_ of 0.42 (ranged from 0.37 to 0.48) and R^2^_CV_ of 0.42 (ranged from 0.1 to 0.65). However, the lowest RMSE_C_ and RMSE_CV_ were found in needle model, with the mean RMSE_C_ of 0.87% (range: 0.82-0.91) and RMSE_CV_ of 0.78% (range: 0.68-0.91), followed by root, branch, generic, and trunk model in sequence. The RMSE_C_ and RMSE_CV_ of the five models were ranging from 0.82 to 1.67 and 0.68 to 1.85, respectively. Very small prediction error was obtained from the 100 simulated models for both individual tissues and generic. The number of latent variables in all of these models is lower than 10. Residual plots showed that all of the individual models and generic models tend to underestimate when *N* values are low and overestimate when *N* is high (Figure 4).

### 3.3. Prediction of *N* Using the NIR Spectra Selected by sMC

**Figure 5:**
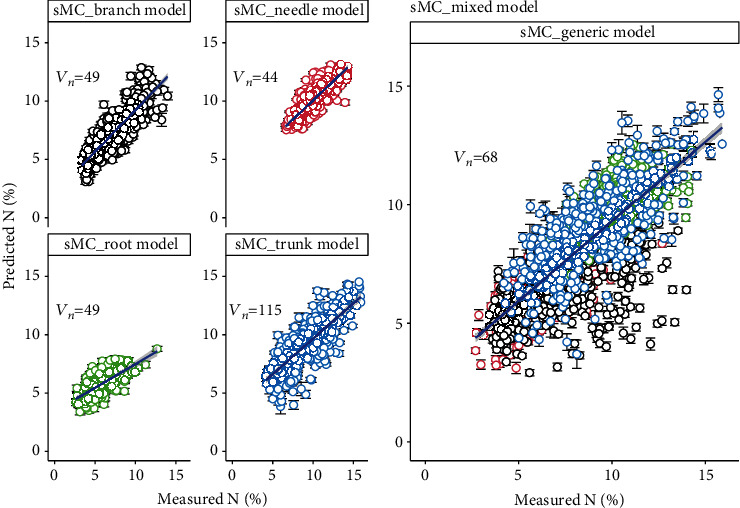
Measured and predicted *N* in the needle, trunk, branch, and root tissues and their generic model using the sMC-selected NIR spectra. Error bars: standard deviations of 100 simulated models. *V*_*n*_: variables selected by sMC selection algorithm.

PLSR models using sMC-selected NIR spectra variables (less than 15% percent of the full length spectra) yield a slightly better results in both individual tissues and generic (Figures 2(b), 2(d), and 5). The mean R^2^_C_ and R^2^_CV_ in models predicting the *N* of both individual and generic tissues were varied from 0.42 to 0.71 and 0.43 to 0.77, respectively. Models predicting the N of branch, needle, trunk, and root had a mean R^2^_C_ of 0.71 (range: 0.69-0.73), 0.67 (range: 0.61-0.71),0.63 (range: 0.591-0.65), and 0.42 (range: 0.37-0.48); mean R^2^_CV_ of 0.77 (range: 0.63-0.86), 0.73 (range: 0.61-0.86),0.71 (range: 0.55-0.86), and 0.43 (range: 0.19-0.65); mean RMSE_C_ of 1.25% (range: 1.20-1.29%), 0.86% (range: 0.79-0.93%), 1.61% (range: 1.54-1.69%), and 1.16% (range: 1.10-1.22%); and mean RMSE_CV_ of 1.23% (range: 0.95-1.43%), 0.78% (range: 0.62-0.91%),1.59% (range: 1.25-1.80%), and 1.15% (range: 0.89-1.47%), respectively. Generic model showed a promising *N* prediction result with high mean R^2^_C_ and R^2^_CV_ of 0.61 (range: 0.59-0.63) and 0.65 (range: 0.56-0.73), and low mean RMSE_C_ and RMSE_CV_ of 1.38 (range: 1.35-1.41) and 1.38 (range: 1.34-1.41), respectively. The sMC models vary larger in the validation set than the calibration set.

### 3.4. Performance Prediction of Individual Tissue Model Applying on Other Tissues

**Figure 6:**
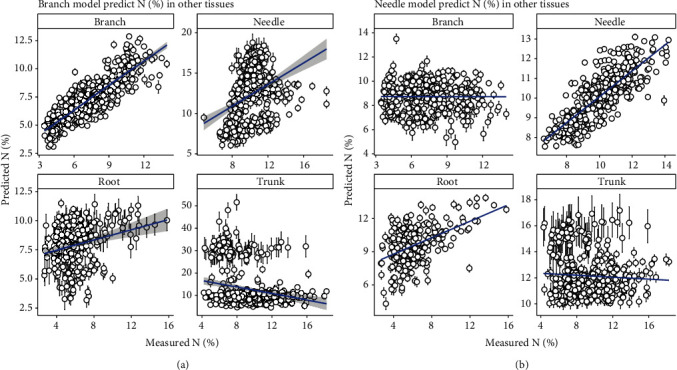
Using individual tissue *N* PLSR model to predict the *N* in other tissues. (a) is using branch model to predict the *N* in four slash pine tissues. (b) is using needle model to predict the *N* in four slash pine tissues.

**Figure 7:**
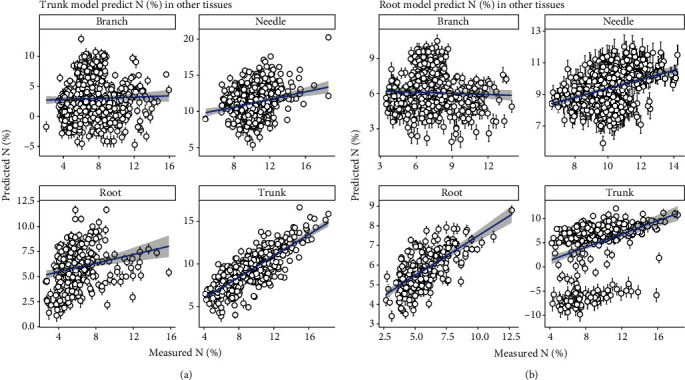
Using individual tissue *N* PLSR model to predict the *N* in other tissues. (a) is using trunk model to predict the *N* in four slash pine tissues. (b) is using root model to predict the *N* in four slash pine tissues.

Individual tissue PLSR model produces a better *N* prediction on their own tissues than on other tissues (Figures 6 and 7). However, generic model, based on both full length NIR spectra and sMC-selected NIR spectra, showed a promising result in predicting *N* of other individual tissues with less bias (Figures 3, 4, and 5).

**Figure 8:**
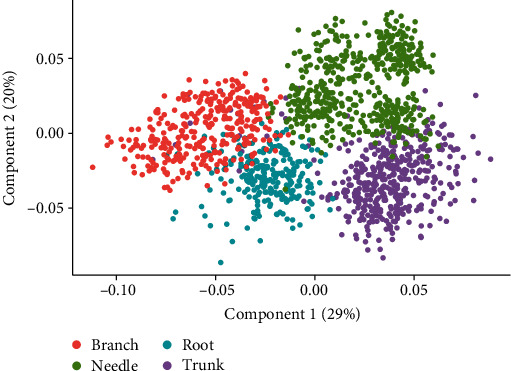
The score plot of the first two components from the mixed tissue sMC_generic model.

Figure 8 is the score plot of sMC_generic model for *N* content prediction. It showed that different types of tissues could be clearly identified by the sMC_generic model, even though the sMC_generic model could efficiently predict the *N* content in all tissues. Branch and root tissues may have similar construction, they are some overlap between them, and needle and most of the trunk are different from branch and root, which have been distributed as a single group, respectively.

### 3.5. Variable Selection of the NIR Spectra Applied in *N* Prediction of Four Tissues

**Figure 9:**
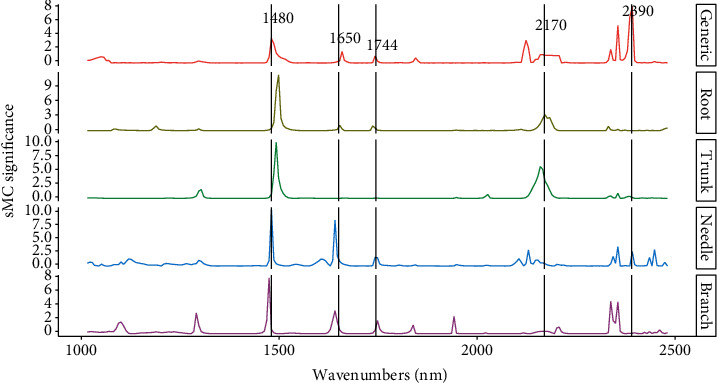
Influence of *N* on NIR spectra in generic, branch, needle, root, and trunk model of slash pine.

Five significantly important regions, i.e., 1650, 1744, 2170, and 2390 nm, were found highly influenced performance of model, both individual and generic. The region around 1480 nm has been recognized as very important among all five models, and the region around 1650 nm has a great significance in needle model than in other models. Regions like 1744 nm were shown to be less important in trunk model than other models. The region around 2390 nm showed a greater significance on the generic and needle model (Figure 9).

## 4. Discussion

In our study, *N* content was variable within different types of tissues. This variation highly affected the model accuracy. It has been reported that less variation of the response characteristics could result in lower model prediction quality [37]. The root *N* content has a low range from 2.7 to 10.2%, and this could explain why the root model produced a lower accuracy of prediction than other models.

*N* is one of the most important macronutrients in plants that strongly influence plant growth and quality. Root in charge with taking up the *N* from the soil in the form of NH4+ and NO3^−^ and mainly stored as proteins, such as ribulose-1, 5-bisphosphate carboxylase/oxygenase (rubisco) [38]. It has been reported that the shortwave infrared region (1300–2500 nm, SWIR) is highly sensitive to plant water, lignin, cellulose, and proteins, and the most frequent mechanism transition between spectra bands and proteins is the N-H stretch, including the 1^st^ and 3^rd^ overtone stretch [39]. There are numerous studies that performed the reflectance data from plant leaves to monitor the *N* traits [40, 41]. However, less is conducted on the different types of tissues [39, 42]. With the high range mean of R^2^_C_ (0.42-0.72) and R^2^_CV_ (0.43-0.77) and low range mean of RMSE_C_ (0.86-1.61%) and RMSE_CV_ (0.78-1.38%) obtained from the individual tissue sMC_(root, branch, needle, and trunk) models and sMC_generic model, our results clearly showed that the *N* content in different types of tissues can accurately and with good level of confidence be predict using reflectance spectroscopy. Furthermore, a generic model can efficiently predict the *N* content in different tissues. In fact, we found that the mean R^2^_C_ of our results in needle model 0.67 (range: 0.61-0.71) was close to the result reported in turfgrass (*R*^2^ = 0.76) leaves [43] and tea leaves (accuracy = 77.3%) [44] but less than that in the olive leaf (*R*^2^ = 0.91) [45] when using the VIS-NIR/NIR spectroscopy.

We found that different individual tissues did perform a reliable prediction PLSR model when we considered the same type of tissues. However, the prediction accuracy extremely decreased when the model was built on one tissue to predict the *N* content in other types of tissues (Figures 6 and 7). Different tissues in trees have different chemical structures, which might result into an absorbance decrease of *N* in the NIR spectra [46]. For example, in the needles, the presence of phenolics or tannins may interfere with the absorbance of *N* content in the NIR spectra and lead to *N* content prediction bias when other types of tissues model are applied. The PLS score plot showed that these four types of tissues can be clearly classified, meaning that besides the *N* content, there are some more chemical or physical information that are different among different tissues, resulting in different absorbance in the specific regions of the NIR spectra.

Similarly, a generic model was made to predict NSC concentration in tree tissues such as root, stem, branch, and leaf of various tree species, and a high prediction accuracy was obtained with *R*^2^ of 0.91 and RMSE of 1.34% [16]. De Bei et al. [47] also found that a robust universal generic model with trunk and leaf tissues of Chardonnay grapevine could be used to predict the total nonstructural carbohydrate (TNC) concentration in both leaves and trunks with a *R*^2^ = 0.86. However, in our study, the accuracy of generic model was slightly low compared to the branch, needle, and trunk model. Thus, it is necessary to build the individual tissue model for *N* content prediction in each tissue themself. Our results reinforced the idea that *N* content prediction in different types of tissues using NIR can be fast and accurate.

It is important to estimate the data distribution and model stability using multiple permutations method [48]. The permutations generated the prediction error (Figures 3, 4, 6, and 7 error bar) based on multiple times of calibration and could be used on the model uncertainty test.

Samples within NIR spectra contain both useful and irrelevant chemical information, and the irrelevant information could reduce the model accuracy. Therefore, it is extremely valuable and useful to extrapolate the key spectra variables to reduce the model calibration error [49]. The sMC method in our study could efficiently select the spectra variables highly related with the *N* content. The sMC_PLSR models for each tree tissues and generic tissues yielded a similar promising result to the full-length spectra with less spectra variables (44 to 115 variables among different tissue models; Figure 4). Additionally, the key wavelengths between 1400-1800 nm and 2100-2400 nm that are highly sensitive to the *N* content were found by sMC method. Important peaks among these regions were found at 1480, 1650, 1744, 2170, and 2390 nm. In our study, the relevant bands that are related to *N* or protein contents are not exactly what other studies have found. A reason for this could be that the different spectral sampling distances from spectral bands vary the number of bands in the spectral range. In addition, different preprocessing methods such as 1st and 2nd derivatives could reduce the band numbers. However, in the context of NIR spectroscopic analysis, the bands sensitive to *N* found in our study can still be found in similar studies. Curran [50] and Kumar et al. [51] both reported that the distinct absorption peaks of proteins and/or *N* are mainly sensitive to the SWIR region which support our study. Spectral bands sensitive to proteins or *N* usually are also related to other components such as lignin (1690 nm and 1940 nm), cellulose (1940 nm), and starch (1690 nm and 1940 nm; [51]. The region around 1650 nm was mainly related to the first overtone of O-H strength which may be dominated by the cellulose in woody samples [52]. Wavelengths around 1480, 1744, 2170, and 2390 nm have been reported to be related to protein contents [47, 50, 53].

## 5. Conclusions

Our results have shown that we can successfully use NIR spectroscopy to characterize the *N* content in tissues of different tree parts. The most important wavelength regions were found by the sMC variable selection method, which contributed to a consistent, promising, and robust calibration model across the four tissues. Our measurement of *N* content in different tissues of trees using NIR spectroscopy is fast and accurate which could highly help for the internal *N* cycling study in the future.

## Data Availability

The data used to support the findings of this study are available from the corresponding author upon request.
